# Exploring Vulnerable, Ethnic Minority, and Low Socioeconomic Children’s Knowledge, Beliefs, and Attitudes Regarding HPV Vaccination in Romania

**DOI:** 10.3390/healthcare13162010

**Published:** 2025-08-15

**Authors:** Teodora Achimaș-Cadariu, Andrei Pașca, Delia Nicoară, Dan Lucian Dumitrașcu

**Affiliations:** 1 Faculty of Medicine, “Iuliu Hațieganu” University of Medicine and Pharmacy, 400012 Cluj-Napoca, Romania; 2 “Prof. Dr. Ion Chiricuta” Institute of Oncology, 400015 Cluj-Napoca, Romania

**Keywords:** vaccine, HPV vaccine, vulnerable population

## Abstract

**Background/Objective:** To assess vulnerable Romanian children’s knowledge, attitudes, and beliefs regarding the HPV vaccination. **Methods:** Vulnerable children (ethnic minorities, high social vulnerability index, or low socioeconomic status) from three schools in Cluj County, Romania, were enrolled in a short educational presentation regarding HPV and were delivered a physical questionnaire consisting of 26 items. **Results:** 199 vulnerable school students concluded the questionnaire with a mean age of 14.62. Most participants were unaware of the HPV infection or the HPV vaccine. Following the educational program, most participants exhibited a reasonably elevated level of knowledge, which positively correlated with the intention to vaccinate. Fifty-three per cent of respondents would vaccinate in school if the vaccine were available, fifty-four per cent would vaccinate if the product were free of charge or at minimal cost, and over sixty-four per cent would vaccinate at their doctor’s recommendation. Several knowledge items, beliefs, and attitudes towards vaccination were disclosed to influence children’s preference to participate in vaccination campaigns. **Conclusions:** This analysis unveiled the pivotal role of knowledge about HPV in the immunization uptake within underserved, vulnerable populations of Romanian children. An intricate interplay between vulnerability, knowledge, accessibility, and the willingness to vaccinate was impacted by several beliefs and attitudes towards HPV vaccination. Most children were willing to participate in HPV immunization campaigns, whether school-based, reimbursed, or at the doctor’s recommendation. These findings act as pillars for assembling future educational campaigns in vulnerable Romanian communities of children, aiming to enhance awareness and coverage of HPV vaccination and ensure inclusive health policies.

## 1. Introduction

Primary prevention has demonstrated its efficacy by reducing health-related diseases, particularly through childhood or adulthood vaccination. Immunization has saved approximately 150 million lives since 1974 [1]. Vaccination programs are targeted at the general population, including children, adolescents, and adults who are at risk or susceptible to developing certain diseases [2].

In 1974, the World Health Organization (WHO) introduced the Expanded Programme on Immunization (EPI), providing universal access to vaccination programs [3]. According to Vanderslott and Marks’s article, only 89 countries have mandatory vaccination policies; 33 countries recommend vaccination, while only 20 have made immunization mandatory for school entry [4]. Despite collective efforts, globally, in 2023, more than 14.5 million children did not receive the recommended childhood vaccinations, boosting the risk of vaccine-preventable outbreaks [5]. Furthermore, newly available vaccines or those with a non-mandatory character express even lower rates. Individual facets contributing to low adherence to vaccination included living in isolated areas, a lack of recommendations from Healthcare Providers (HCPs), low awareness and education, being part of a migrant population, and having a low socioeconomic status [6,7].

Cervical cancer is among the few preventable oncological pathologies for which efficacious primary prophylaxis methods, such as vaccination, are well documented. The Human Papillomavirus (HPV) vaccine is highly effective in preventing infections and, consequently, cervical cancer (CC). It was first approved by the Food and Drug Administration (FDA) in 2006 [8]. Despite being available for almost 2 decades, the coverage remains low. In 2023, only 15% of girls aged 15 years old were fully vaccinated globally, while for boys, this percentage was estimated to be below 5% [9]. Hence, CC remains a noteworthy public health issue due to its high incidence (662.044 cases) and mortality (348.709 deaths as of 2022) [10].

It has formerly been uncovered that communities with a high social vulnerability index or a lower density of available doctors express lower chances of immunization [11]. Child poverty and Health Care Practitioner (HCP) shortage were negatively associated with the initiation and completion of HPV vaccination schemes in both male and female children, contributing to vaccine hesitancy [12].

In Romania, the HPV vaccine was first implemented in 2008 as a school-based vaccination program or accessed through family doctors, targeting girls aged 10–11. The campaign was briefly interrupted due to deficient coverage, reaching around 2% of the targeted population. It was relaunched in 2009, targeting girls aged 12–14, with modest developments. In 2023, the HPV immunization was incorporated into the National Immunization Program as a gender-neutral vaccine conceived for children and teenagers aged 11 to 18 years old. It was offered free of charge or partially reimbursed (50%) for women aged 18 or older [13]. In 2025, the free vaccination schedule was expanded to individuals aged up to 26 years old. According to the World Health Organization (WHO), in 2023, the HPV vaccination program coverage was approximately 6% for girls aged 15, while no information was available regarding boys [9]. At the European level, Romania is nowadays ranking first in incidence and mortality attributed to CC with an Age-Standardized Rate (ASR) of 21.7/100,000 and an ASR in mortality of 9.3/100,000 [14,15].

Vulnerable populations contribute to somber statistics regarding vaccination rates, with lower vaccination coverage rates uncovered among them. Nevertheless, several exploratory analyses have demonstrated the cost-effectiveness of vaccinating socially vulnerable individuals [16]. Prior analyses have delivered valuable insights into potential interventions addressing the low uptake of the HPV vaccine, such as school-based vaccination campaigns to which hard-to-reach populations have been responsive [17]. Knowledge was also emphasized as a definitive characteristic associated with initiation and completion of the HPV vaccination schemes among vulnerable individuals [18]. Therefore, it is crucial to address both system-level barriers and individual characteristics in vulnerable populations to enhance immunization coverage. The present study sought to identify factors that could influence vulnerable children and adolescents to vaccinate against HPV, considering the costs, doctors’ recommendations, and school-based vaccination in Romania. This will address a present research gap in investigating knowledge, beliefs, and attitudes towards HPV vaccination in vulnerable Romanian children, stemming from poverty or ethnic minority communities. This will serve as foundational research for assembling educational campaigns in vulnerable Romanian communities of children, aiming to enhance the low awareness and coverage of HPV vaccination and ensure inclusive health policies.

## 2. Materials and Methods

### 2.1. Participants

The sample consisted of vulnerable children (ethnic minorities, high social vulnerability index, or low socioeconomic status) from three schools in Cluj County, Romania. Low socioeconomic index was defined by lower income (per household), limited educational attainment, lower status occupations, living in disadvantaged areas, etc. High social vulnerability was assessed through socioeconomic status, minority status, difficulty in accessing education or healthcare services, etc. The participant schools were recruited with the aid of the School Inspectorate, which identified at-risk populations. These schools were situated in isolated or poor areas, and most of the attending children were part of the targeted populations. Meetings were undertaken with the schools’ principals and class conductors to ensure all participants were eligible. In addition to selecting participants, the class conductors facilitated a meeting with the parents of minors. One investigator (D.N.) attended a teacher-parent meeting where the study objectives and methods were explained. During the meeting, verbal informed consent was obtained from the parents of minors. Only children whose parents (or guardians) agreed to participate were enrolled in the study. Data were collected in 2022, following approval from the Ethics Committee of Prof. Dr. Ion Chiricuta Institute of Oncology, Cluj-Napoca, Romania, under number 234 on 17 February 2022. It also offered a waiver for the written consent. The rationale for this simplified procedure was reached after careful consultation with the School Inspectorate. It was established that a significant part of parents had lower educational levels that would not enable them to properly read and understand the study’s objectives and methods, nor to accurately express written informed consent. Therefore, it was decided that verbal consent was the simplest and effective way to reach these vulnerable populations.

### 2.2. Instruments

The participants were verbally asked questions about their awareness and knowledge regarding HPV infection and the HPV vaccine. After that, a presentation was delivered that lasted approximately 50 min (incorporated into the school’s program) and included information about vaccines in general, HPV infection, and preventive methods, such as vaccination, along with their importance. One of the investigators (D.N), a public health specialist, delivered the presentation under the surveillance of the class conductors. Only minors whose parents or guardians agreed to participate were involved in the study. No pre-presentation assessment was conducted as the purpose of the study was not to evaluate the efficacy of a school-based educational intervention, but rather to provide all students with a knowledge baseline before assessing their willingness to vaccinate in various scenarios.

Following the educational intervention, a questionnaire (Supplementary File S1) was administered to assess their knowledge, beliefs, and intention to vaccinate against HPV. The questionnaire was delivered in a physical form. It included 26 questions split into categories: demographic questions, knowledge regarding HPV, perceived efficacy of vaccines, and attitudes towards vaccination, offering responses on a Likert scale (1—strongly disagree, 2—disagree, 3—not sure, 4—somewhat agree, 5—strongly agree).

### 2.3. Data Analysis

The data analysis was conducted using JASP Statistics software [19]. The Likert scale was transformed as follows: values of 1 or 2 were transformed into No/disagree answers, three were considered neutral/not sure, while 4 or 5 were considered Yes/agree. For the statistical analysis, the Chi-squared test was used to assess the correlation between categorical variables, with a 95% Confidence Interval (CI) and a *p*-value threshold of 0.05. The Chi-square statistic is a non-parametric test for nominal values. It presents significant advantages like bypassing the need for equality of variances in study populations. However, it only shows the significance of the results and never the strength of association. Therefore, Cramer’s V was deployed to address this, as an indicator for power analysis [20]. The measure of strength was performed considering 4 degrees of freedom (df) and respective values for Cramer’s V as follows: 0.05 highlighted a small effect size, 0.15 a medium, and 0.25 a large effect size [21].

## 3. Results

One hundred ninety-nine vulnerable school students (ethnic minorities and those from low socioeconomic backgrounds) completed the questionnaire, with a mean age of 14.628 (SD = 2.594).

Most participants were not aware of or did not know about the HPV infection or the HPV vaccine. Following the educational intervention, most participants demonstrated a reasonably high level of knowledge, as indicated in Table 1.

### 3.1. HPV Knowledge and the Intention to Vaccinate

Basic knowledge has been statistically correlated with the intention to vaccinate. Over one hundred participants knew that HPV infection could cause cervical cancer and would like to get vaccinated, regardless of the school-based implementation, a doctor’s recommendation, or whether the vaccines would be free of charge. Knowledge regarding the causality of genital warts and the HPV being classified as an STD was also statistically correlated with the intention to vaccinate, regardless of the implementation method (free of charge, school-based, or at a doctor’s recommendation). Further data can be consulted in Table 2.

The intention to vaccinate at free cost was not statistically significant among those who knew that HPV could cause infertility. Knowledge regarding the use of condoms did not seem to influence vaccination rates at the doctor’s recommendation. Moreover, believing that the HPV infection is asymptomatic did not correlate with the willingness to vaccinate in school-based programs.

Regarding free-of-charge vaccination, a large effect size was identified regarding knowledge that the HPV infection is an STD, condoms are effective in preventing the HPV infection, the HPV infection can cause genital warts, and the HPV can cause cervical cancer. A medium effect size was shown in that the infection can be asymptomatic.

For the doctor’s recommendation, large power effects were highlighted in the following variables: the HPV infection is an STD, the HPV infection is asymptomatic, the HPV infection can cause genital warts, and the HPV infection can cause cervical cancer. A medium effect was identified in only one variable: the HPV infection can cause infertility.

Finally, when asked about school-based programs, large and medium effect sizes were shown in the analysis, ranging from as low as Cramer’s V = 0.142 for the infection as being asymptomatic to Cramer’s V = 0.333 for knowing that the HPV infection causes genital warts.

### 3.2. Beliefs About Immunization, Perceived Efficacy, and the Willingness to Vaccinate Against HPV

Positive beliefs correlated with the intention to vaccinate in all given situations: free of charge, at the doctor’s recommendation, or in school-based programs. A total of 117 children believed that vaccines could prevent infection by others. The majority of participants trusted older vaccines (55.77%), while almost 70% believe that vaccines get better over time. Interestingly, 41 participants (20%) believed that administering the HPV vaccine to children is like experimenting on them. The opposite belief correlated with the intention to vaccinate. Results highlight the importance of positive beliefs regarding HPV vaccination and the need for perceived efficacy. Detailed results are shown in Table 3.

### 3.3. Attitudes Towards HPV Immunization

Concerning the possibility of finding a sexual partner if diagnosed with condylomas, almost 34% (68) expressed worries. School-based vaccination intention was not correlated with worrying about finding sexual partners. Interestingly, 87 participants believed that they should be vaccinated without parental consent (almost 44%). Strong correlation with above threshold values for Cramer’s V statistics was observed in these groups, regardless of the proposed method for obtaining the vaccine. A total of 11% (22) of participants stated that they would prefer not to get vaccinated because of the pain involved in the process. Moreover, 34 respondents (more than 17%) stated that they would only vaccinate against HPV if the vaccine were to become mandatory. More than 40% (81) of children were unsure of the vaccinated status of their closest friends. Table 4 presents detailed statistics.

When free of charge, significant effects were seen in receiving the HPV vaccine without parental consent (Cramer’s V = 0.262), painful vaccination (Cramer’s V = 0.285), the non-compulsory character of the HPV vaccine (Cramer’s V = 0.303), friends are vaccinated (Cramer’s V = 0.315), doctor’s recommendation (Cramer’s V = 0.453), and receiving the HPV vaccine in school (Cramer’s V = 0.51).

When asked about willingness to vaccinate at a doctor’s recommendation, large effects were seen in the analysis, ranging from Cramer’s V = 0.252 to 0.505. Similar findings were highlighted for proposed school-based vaccination, where only believing that friends were vaccinated against HPV showed a medium effect size, with Cramer’s V = 0.248, while the remaining variables showed a large effect size.

### 3.4. Gender Comparison

A total of 94 boys and 105 girls completed the survey, with a median age of 14. The mean age of girls was 14.81 (SD = 3.3, IQR = 1). The mean age of boys was 14.42 (SD = 1.41, IQR = 1).

Most girls and boys stated that they would vaccinate if the vaccine was free or at a low cost, with no statistically significant difference (X^2^ (2, N = 193) = 2.828, *p* = 0.243). There was also no statistical difference between boys and girls in vaccination intent if they would receive a doctor’s recommendation, X^2^ (2, N = 191) = 0.534, *p* = 0.766, or if the HPV vaccination would be offered in school, X^2^ (4, N = 193) = 0.878, *p* < 0.645. Knowledge about the causality of Cervical Cancer was statistically significantly different between genders. Elaborated details can be found in Table 5.

## 4. Discussion

Scarce data are available regarding the knowledge and beliefs of vulnerable populations in Romania regarding HPV infection or immunization. The present analysis is among the first investigating the intention of vulnerable Romanian children (ethnic minorities or low socioeconomic status) to vaccinate against HPV. This initiative took into consideration the costs, the doctor’s recommendation, and potential school-based vaccination programs. Many variables were correlated with vaccination intention, including knowledge, perceived effectiveness of vaccines, and positive attitudes toward vaccination. Of the aforementioned, knowledge was demonstrated to have a substantial impact on one’s attitudes regarding immunization [22]. Iova’s scoping review pinpointed that vaccination uptake increased following educational interventions [23]. Further proving the role of knowledge in the decision-making process, an investigation of Romanian women also revealed that although more than 62% of respondents were aware of the HPV vaccine, their knowledge was minimal or incomplete; only half of them had a positive attitude towards immunization [24]. Moreover, in the ethnic minority groups, the level of awareness is significantly lower compared to the general population. Investigations uncovered that up to 70% of women were unaware of the HPV infection [25]. Other studies highlighted a cultural aversion against immunization and HPV vaccines within certain ethnical minority communities like Roma [26]. Little data were published regarding Romanian adolescents’ knowledge of HPV. In the Voidăzan et al. study, around 50% of children had poor knowledge about the HPV infection, and up to 70% of them had poor to very poor self-assessed knowledge about immunization [27]. Another investigation uncovered low knowledge and awareness regarding the HPV vaccines among general teen populations in northwest Romania, with percentages unaware of the vaccine varying from 19% to almost 37% [28]. Consequently, very low vaccination rates were ascertained in preceding publications, as low as 2.3%. In 80% of cases, the main reason for not attending immunization was the lack of information [29].

No data were identified in the literature regarding vulnerable children or adolescents from ethnic minority backgrounds, low socioeconomic status, or high social vulnerability indexes. Interestingly, a previous publication [30] suggested that a sample of the Roma population from Hungary did not behave differently from the general population regarding screening attendance. The publication also suggested that, rather than ethnic considerations, previous negative experiences with the healthcare system were significant determinants in vaccination adherence. Nevertheless, the study only included Roma women with permanent addresses, potentially skewing the results and generalizability. Lower rates for CC screening were also observed in Hungarian Roma women compared to the non-Roma population [31]. A Slovakian study also uncovered impaired relationships between the Roma population and health care providers, which led to significant avoidance of health services, hindering HPV vaccination [32]. The present analysis encompasses 199 vulnerable children from these groups, delivers an educational presentation, and assesses consequent knowledge, beliefs, and attitudes towards the HPV vaccination. Even after a short educational presentation about HPV, only around 50% of respondents understood that HPV can cause CC, while the other half were unsure or unaware. Less than half of the participants were aware that the HPV infection could be asymptomatic.

No statistically significant differences were highlighted between male and female respondents apart from knowledge about HPV and the etiopathogenesis of CC. More than 80% of boys knew that HPV could cause CC, whereas only 63% of girls were aware of the causality. Statistical significance was observed with a Chi-square value of just above 6 and a *p* of 0.046, although not strongly correlated with Cramer’s V values of 0.177.

Only 53% to 54% of children would vaccinate against HPV if the vaccine were free of charge or at minimal cost, whereas more than 64% would partake in immunization if their doctors recommended it. These findings suggest that even when specific barriers, such as financial burdens or low accessibility, are eliminated, a doctor’s recommendation will potentially provide more weight in the vulnerable populations’ decision to vaccinate against HPV. Another interesting finding pertains to children’s beliefs regarding parental consent for vaccination. A total of 87 respondents (or over 43%) believed that teenagers should be vaccinated against HPV without parental consent. Previous investigations have uncovered several characteristics that lead to vaccine hesitancy among European parents [33]. Although not the primary focus of the current analysis, hypothetical interventions such as school-based vaccination programs can be beneficial in overcoming hesitancy among parents in vulnerable populations and potentially bypassing negative consent of parents.

Peer influence could also deter young adolescents from getting vaccinated against HPV. A total of 40% of respondents from the present investigation were unsure about their closest friends’ vaccination status, highlighting hesitancy in communicating about HPV or the HPV vaccine. As part of the peer influence, neighborhood sociodemographic factors were significant determinants of vaccination among ethnic minorities and low-income girls [34]. Behavioral, normative, and control beliefs were predictors for vaccination initiation in economically disadvantaged women [35]. All these social and demographic factors contribute to a complex interaction between background, knowledge, peer pressure, accessibility, and the desire to vaccinate against HPV. In disadvantaged educational settings, care must be taken to address peer pressure and misinformation, as previous articles have uncovered lower vaccination rates due to disinformation within school-based campaigns [36].

### 4.1. Limitations

The small sample size represents a limitation of the current analysis. However, it is essential to acknowledge the inclusion of hard-to-reach populations in the analysis. This potentially provides valuable insights into the hindrances to vaccination against HPV that these groups face, regardless of the sample size. Sound statistical inferences were drawn as enough data were provided.

Another issue is represented by the geographical limitations of the recruited participants. All 199 children were originating from the county of Cluj, Romania. This is due to the fact that all investigators were based in the region, thus facilitating the ease of access in these vulnerable, hard-to-reach communities. As such, the school inspectorate aided in selecting designated schools in isolated or poor regions, where the majority of attending children were eligible for inclusion. As part of recent endeavors, vaccination and mobile screening campaigns have been undertaken in isolated, poor areas with the aid of a tertiary cancer center in the area. The current investigation was an initial exploratory analysis that strived to provide essential knowledge and assess the opportunity for future school-based vaccination campaigns or educational initiatives.

The lack of a pre-intervention assessment might represent another limitation. Even if not directly recorded, information regarding the vaccinal status of the children was obtained through the school general practitioner (GP) and measured at below 2%, making statistical inferences irrelevant. Moreover, it was not the aim nor the objective of the present analysis to assess vaccination rates among vulnerable children. This was previously ascertained in other investigations. It is important to acknowledge that the present investigation did not strive to evaluate an educational intervention among vulnerable children, but rather to assess factors influencing the willingness to vaccinate, while offering every student a knowledge baseline.

The absence of a control group limits the generalizability of the results. It was not the purpose of this investigation to compare vulnerable children to students originating from higher-developed areas, but rather to assess factors influencing decisions to vaccinate in this population. Also limiting the results is the lack of subgrouping based on the type of vulnerability. No data were collected to ascertain the apprehension of a subgroup (ethnic minority, low socioeconomic status, poverty, isolated areas). This was due to the fact that most children involved in the study had at least one or two of the characteristics, rendering categories as superposable and potential subgroup analyses less relevant.

### 4.2. Future Directions

Although not within the scope of this investigation, the educational program delivered showed high efficacy in enhancing participants’ knowledge regarding HPV-related diseases. The need for an effective information campaign is strong, and future research must target vulnerable populations specifically. Findings suggest a complex interplay between the lack of knowledge, peer influence, parental consent, misinformation about HPV, and the willingness to vaccinate. Several solutions may be deployed to ensure inclusive vaccination tactics enrich knowledge and increase uptake in these populations. Care must be taken when developing educational programs, as various structures should be involved in designing and delivering them. Perhaps training specialists in culturally sensitive communication methods could enhance deliverability. On the other hand, positive results might be achieved through peer-to-peer education in low-income settings [37] or engaging members from underserved communities in designing and optimizing immunization interventions tailored to vulnerable populations [38].

The role of HCPs was previously determined as paramount in increasing vaccination norms [39,40]. Often, school doctors, GPs, or family doctors are the only medical contact for many vulnerable children or adolescents. A study on local populations from Cluj-Napoca investigated vaccine hesitancy in more than 450 participants and found that HPV vaccines were among the most common types of vaccines producing hesitancy. One of the main reasons for declining immunization for their children among parents was negative media coverage [41]. Mass media coverage was found to be potentially inaccurate or less likely to provide comprehensive data [42], while social media platforms may deter populations from HPV vaccination [43].

School-based vaccination is another feasible option to increase immunization. More than 53% of the vulnerable children would be vaccinated in school if they could get the vaccine. Interventions of this nature were deemed successful and proved to increase vaccination coverage in vulnerable populations of low-income countries [44]. Moreover, similar positive effects were highlighted in high-income countries, with higher benefits in schools with lower socioeconomic positions, deeming the campaigns highly suitable in these environments [45]. A systematic review and meta-analysis proved that school-based vaccination was highly efficient in increasing uptake [46], and alternative interventions proved beneficial even in reducing vaccine-related pain, anxiety, or fear [47]. Moreover, leveraging health infrastructure to enhance coverage is beneficial in increasing uptake, especially in hard-to-reach areas where combining vaccination campaigns is an opportunity to immunize vulnerable populations [48].

Although it might appear paradoxical, studying HPV vaccination beliefs and practices among vulnerable children in Romania has sound considerations. Romania ranks among the first European countries in CC incidence and mortality, and vaccination rates remain unremarkable. Nevertheless, the country has recently begun to develop vaccination strategies and is in the early stages of developing a screening program for CC. With the development of a National Plan for Combating Cancer, HPV vaccination became a priority milestone. While conceiving regional or even national endeavors to increase the immunization uptake, underserved communities must be included early on. Therefore, the present analysis might serve as a foundational insight for public health policymakers in Romania that will aid in developing inclusive vaccination campaigns that reach out to vulnerable children and ensure inclusive tactics.

## 5. Conclusions

The present analysis uncovered the pivotal role of knowledge in immunization uptake within underserved, vulnerable populations of Romanian children. A complex interplay between vulnerability, knowledge, accessibility, and the willingness to vaccinate was influenced by several beliefs and attitudes towards HPV vaccination. Most children were willing to participate in immunization campaigns, whether school-based, reimbursed, or recommended by a doctor. These findings serve as pillars for constructing informational and educational campaigns in vulnerable Romanian communities of children. Those efforts should aim to improve the low awareness and coverage of the HPV vaccination and ensure inclusive health policies.

## Figures and Tables

**Table 1:** HPV knowledge.

Variable	Answer	Frequency	Percentage
The HPV infection is an STD	No	4	2.01
	Not sure	36	18.09
	Yes	159	79.89
The HPV infection can be asymptomatic	No	60	3.151
	Not sure	50	25.12
	Yes	89	44.72
The use of condoms can prevent the HPV infection	No	52	26.13
	Not sure	80	40.20
	Yes	67	33.66
The HPV infection can cause infertility	No	68	34.17
	Not sure	64	32.16
	Yes	67	33.66
HPV can cause genital warts or condylomas	No	16	8.04
	Not sure	50	25.64
	Yes	129	64.82
HPV can cause Cervical Cancer	No	54	27.13
	Not sure	43	21.60
	Yes	102	51.25

**Table 2:** HPV knowledge and the willingness to vaccinate.

	Free of Charge Vaccination			Doctor’s Recommendation			School-Based Vaccination		
Variable	Chi^2^	*p*-Value	Cramer’s V	Chi^2^	*p*-Value	Cramer’s V	Chi^2^	*p*-Value	Cramer’s V
The HPV infection is an STD	36.78	<0.001	0.30	35.05	<0.001	0.30	39.26	<0.001	0.31
The HPV infection can be asymptomatic	20.84	<0.001	0.23	24.61	<0.001	0.25	7.82	0.098	0.14
The use of condoms can prevent the HPV infection	25.55	<0.001	0.25	8.91	0.063	0.15	18.58	<0.001	0.21
The HPV infection can cause infertility	7.51	0.111	0.14	19.45	<0.001	0.22	11.84	0.019	0.17
Genital warts or condylomas can be caused by HPV	53.10	<0.001	0.37	39.54	<0.001	0.32	41.84	<0.001	0.33
HPV can cause Cervical Cancer	75.08	<0.001	0.44	69.09	<0.001	0.42	36.14	<0.001	0.30

**Table 3:** HPV beliefs, perceived efficacy, and the willingness to vaccinate.

	Free of Charge Vaccination			Doctor’s Recommendation			School-Based Vaccination		
Beliefs/Perceived Efficacy	Chi^2^	*p*-Value	Cramer’s V	Chi^2^	*p*-Value	Cramer’s V	Chi^2^	*p*-Value	Cramer’s V
Mandatory vaccines can prevent children from getting infected by unvaccinated children	39.92	<0.001	0.32	52.75	<0.001	0.37	75.42	<0.001	0.44
I understand what the HPV vaccine is used for	48.62	<0.001	0.35	70.90	<0.001	0.43	38.05	<0.001	0.31
I’m more likely to trust older vaccines	55.33	<0.001	0.38	59.04	<0.001	0.39	41.99	<0.001	0.33
Vaccines get better because of research	74.36	<0.001	0.43	71.18	<0.001	0.43	63.21	<0.001	0.40
Children should only be vaccinated against serious diseases (N)	37.13	<0.001	0.31	49.56	<0.001	0.36	32.17	<0.001	0.28
Healthy children do not need to be vaccinated (N)	42.35	<0.001	0.33	48.14	<0.001	0.35	26.63	<0.001	0.26
An HPV vaccine could prevent future health issues	83.19	<0.001	0.46	57.03	<0.001	0.38	36.28	<0.001	0.30
Administering the HPV vaccine for children is like experimenting on them (N)	21.19	<0.001	0.23	35.55	<0.001	0.30	22.19	<0.001	0.24
Most of the people I know believe that the HPV vaccine before adolescence is a good idea	94.64	<0.001	0.49	57.37	<0.001	0.38	30.20	<0.001	0.28

**Table 4:** Attitudes towards vaccination and the willingness to vaccinate.

	Free of Charge Vaccination			Doctor’s Recommendation			School-Based Vaccination		
Variable	Chi^2^	*p*-Value	Cramer’s V	Chi^2^	*p*-Value	Cramer’s V	Chi^2^	*p*-Value	Cramer’s V
Genital warts or condylomas can make it harder to find a sexual partner	21.73	<0.001	0.24	29.73	<0.001	0.28	8.67	0.07	0.15
A teenager should be vaccinated against HPV without parental consent	26.4	<0.001	0.26	64.09	<0.001	0.41	83.06	<0.001	0.46
Vaccine are very painful so I would prefer not to vaccinate against HPV (N)	30.92	<0.001	0.28	58.41	<0.001	0.39	52.49	<0.001	0.37
I will not vaccinate if the HPV vaccine is not mandatory (N)	35.36	<0.001	0.30	37.72	<0.001	0.31	38.90	<0.001	0.31
I will vaccinate against HPV regardless of the costs	48.10	<0.001	0.35	97.06	<0.001	0.50	81.64	<0.001	0.46
All my closest friends are vaccinated against HPV	38.11	<0.001	0.31	24.29	<0.001	0.25	23.61	<0.001	0.24
I would vaccinate against HPV if my doctor would recommend it	78.21	<0.001	0.45				89.86	<0.001	0.48
I would vaccinate if it was free of charge or with very little costs				78.21	<0.001	0.45	100.52	<0.001	0.51
I would vaccinate against HPV if I could get the vaccine in school	100.52	<0.001	0.51	89.86	<0.001	0.48			

**Table 5:** Gender comparison. Bold text highlights statistical significance.

	Statistics		
Variable	Chi^2^	*p*-Value	Cramer’s V
The HPV infection is an STD	3.40	0.182	0.13
The HPV infection can be asymptomatic	3.98	0.137	0.14
The use of condoms can prevent the HPV infection	4.10	0.128	0.14
The HPV infection can cause infertility	0.66	0.717	0.05
Genital warts or condylomas can be caused by HPV	3.10	0.212	0.12
**HPV can cause Cervical Cancer**	**6.14**	**0.046**	**0.17**
Mandatory vaccines can prevent children from getting infected by unvaccinated children	4.68	0.096	0.15
I understand what the HPV vaccine is used for	1.99	0.368	0.10
Genital warts or condylomas can make it harder to find a sexual partner	1.68	0.43	0.09
I’m more likely to trust older vaccines	1.39	0.497	0.08
Vaccines get better because of research	4.97	0.083	0.15
Children should only be vaccinated against serious diseases	5.47	0.065	0.16
Healthy children do not need to be vaccinated	0.49	0.781	0.05
An HPV vaccine could prevent future health issues	1.66	0.434	0.09
Administering the HPV vaccine for children is like experimenting on them	4.89	0.086	0.15
Most of the people I know believe that the HPV vaccine before adolescence is a good idea	2.73	0.254	0.11
A teenager should be vaccinated against HPV without parental consent	0.50	0.775	0.05
Vaccine are very painful so I would prefer not to vaccinate against HPV	5.7	0.058	0.17
I will not vaccinate if the HPV vaccine is not mandatory	3.71	0.205	0.12
I will vaccinate against HPV regardless of the costs	0.47	0.79	0.05
All my closest friends are vaccinated against HPV	4.14	0.126	0.14
I would vaccinate against HPV if my doctor would recommend it	0.53	0.776	0.05
I would vaccinate against HPV if it was free of charge or with very little costs	2.82	0.243	0.12
I would vaccinate against HPV if I could get the vaccine in school	0.87	0.645	0.06

## Data Availability

The data presented in this study are available on request from the corresponding author. Data available on request due to restrictions.

## Supplementary Materials

The following supporting information can be downloaded at: https://www.mdpi.com/article/10.3390/healthcare13162010/s1. Supplementary File S1: A questionnaire; Table S1: HPV-related knowledge; Table S2: Gender comparison.
