# Supplementary material for: Exploring Vulnerable, Ethnic Minority, and Low Socioeconomic Children’s Knowledge, Beliefs, and Attitudes Regarding HPV Vaccination in Romania
Source: Healthcare (Basel). 2025 Aug 15;13(16):2010. doi: 10.3390/healthcare13162010 (PMC12385608; doi:10.3390/healthcare13162010)
Supplement: Supplementary file 1 [file healthcare-13-02010-s001.zip › healthcare-3741958-supplementary.pdf]

Supplementary File 1: A Questionnaire

1. Gender

Male

Female

Age

(open-ended)

1. The HPV infection is an STD

1 – strongly disagree 2 – disagree 3 - not sure 4 - somewhat agree 5 – strongly agree

2. The HPV infection can be asymptomatic

1 – strongly disagree 2 – disagree 3 - not sure 4 - somewhat agree 5 – strongly agree

3. The use of condoms can prevent the HPV infection

1 – strongly disagree 2 – disagree 3 - not sure 4 - somewhat agree 5 – strongly agree

4. The HPV infection can cause infertility

1 – strongly disagree 2 – disagree 3 - not sure 4 - somewhat agree 5 – strongly agree

5. Genital warts or condylomas can be caused by HPV

1 – strongly disagree 2 – disagree 3 - not sure 4 - somewhat agree 5 – strongly agree

6. HPV can cause Cervical Cancer

1 – strongly disagree 2 – disagree 3 - not sure 4 - somewhat agree 5 – strongly agree

7. Mandatory vaccines can prevent children from getting infected by unvaccinated children

1 – strongly disagree 2 – disagree 3 - not sure 4 - somewhat agree 5 – strongly agree

8. I understand what the HPV vaccine is used for

1 – strongly disagree 2 – disagree 3 - not sure 4 - somewhat agree 5 – strongly agree

9. Genital warts or condylomas can make it harder to find a sexual partner

1 – strongly disagree 2 – disagree 3 - not sure 4 - somewhat agree 5 – strongly agree

10. I'm more likely to trust older vaccines

1 – strongly disagree 2 – disagree 3 - not sure 4 - somewhat agree 5 – strongly agree

11. Vaccines get better because of research

1 – strongly disagree 2 – disagree 3 - not sure 4 - somewhat agree 5 – strongly agree

12. Children should only be vaccinated against serious diseases

1 – strongly disagree 2 – disagree 3 - not sure 4 - somewhat agree 5 – strongly agree

13. Healthy children do not need to be vaccinated

1 – strongly disagree 2 – disagree 3 - not sure 4 - somewhat agree 5 – strongly agree

14. An HPV vaccine could prevent future health issues

1 – strongly disagree 2 – disagree 3 - not sure 4 - somewhat agree 5 – strongly agree

15. Administering the HPV vaccine for children is like experimenting on them

1 – strongly disagree 2 – disagree 3 - not sure 4 - somewhat agree 5 – strongly agree

16. Most of the people I know believe that the HPV vaccine before adolescence is a good ideea

1 – strongly disagree 2 – disagree 3 - not sure 4 - somewhat agree 5 – strongly agree

17. A teenager should be vaccinated against HPV without parental consent

1 – strongly disagree 2 – disagree 3 - not sure 4 - somewhat agree 5 – strongly agree

18. Vaccine are very painful so I would prefer not to vaccinate against HPV

1 – strongly disagree 2 – disagree 3 - not sure 4 - somewhat agree 5 – strongly agree

19. I will not vaccinate if the HPV vaccine is not mandatory

1 – strongly disagree 2 – disagree 3 - not sure 4 - somewhat agree 5 – strongly agree

20. I will vaccinate against HPV regardlessof the costs

1 – strongly disagree 2 – disagree 3 - not sure 4 - somewhat agree 5 – strongly agree

21. All my closest friends are vaccinated against HPV

1 – strongly disagree 2 – disagree 3 - not sure 4 - somewhat agree 5 – strongly agree

22. I would vaccinate against HPV if my doctor would recommed it

1 – strongly disagree 2 – disagree 3 - not sure 4 - somewhat agree 5 – strongly agree

23. I would vaccinate against HPV if it was free of charge or with very little costs

1 – strongly disagree 2 – disagree 3 - not sure 4 - somewhat agree 5 – strongly agree

24. I would vaccinate against HPV if I could get the vaccine in school

1 – strongly disagree 2 – disagree 3 - not sure 4 - somewhat agree 5 – strongly agree

Table S1. HPV-related knowledge

	Free of charge or low cost			Doctor's recommendation			School-Based		
Variable	Chi2	p	Cramer's V	Chi2	p	Cramer's V	Chi2	p	Cramer's V
The HPV infection is an STD	36.782	< 0.001	0.309	35.053	< 0.001	0.303	39.264	< 0.001	0.319
The HPV infection can be asymptomatic	20.848	< 0.001	0.232	24.614	<.001	0.254	7.829	0.098	0.142
The use of condoms can prevent the HPV infection	25.559	< 0.001	0.257	8.91	0.063	0.153	18.585	< 0.001	0.219
The HPV infection can cause infertility	7.516	0.111	0.14	19.458	< 0.001	0.226	11.843	0.019	0.175
Genital warts or condylomas can be caused by HPV	53.104	< 0.001	0.375	39.546	< 0.001	0.323	41.848	< 0.001	0.333
HPV can cause Cervical Cancer	75.081	< 0.001	0.445	69.099	< 0.001	0.426	36.145	< 0.001	0.308
Mandatory vaccines can prevent children from getting infected by unvaccinated children	39.926	< 0.001	0.324	52.754	< 0.001	0.373	75.426	< 0.001	0.446
I understand what the HPV vaccine is used for	48.62	< 0.001	0.359	70.908	< 0.001	0.435	38.05	< 0.001	0.317
Genital warts or condylomas can make it harder to find a sexual partner	21.731	< 0.001	0.241	29.735	< 0.001	0.283	8.67	0.07	0.152
I'm more likely to trust older vaccines	55.332	< 0.001	0.382	59.04	< 0.001	0.396	41.99	< 0.001	0.332
Vaccines get better because of research	74.362	< 0.001	0.439	71.185	< 0.001	0.432	63.217	< 0.001	0.405
Children should only be vaccinated against serious diseases	37.133	< 0.001	0.311	49.567	< 0.001	0.36	32.179	< 0.001	0.289
Healthy children do not need to be vaccinated	42.356	< 0.001	0.331	48.146	< 0.001	0.355	26.633	< 0.001	0.263
An HPV vaccine could prevent future health issues	83.191	< 0.001	0.467	57.032	< 0.001	0.386	36.286	< 0.001	0.308
Administering the HPV vaccine for children is like experimenting on them	21.198	< 0.001	0.236	35.559	< 0.001	0.306	22.195	< 0.001	0.241
Most of the people I know believe that the HPV vaccine before adolescence is a good idea	94.647	< 0.001	0.496	57.372	< 0.001	0.388	30.207	< 0.001	0.28
A teenager should be vaccinated against HPV without parental consent	26.4	< 0.001	0.262	64.096	< 0.001	0.41	83.06	< 0.001	0.465
Vaccine are very painful so I would prefer not to vaccinate against HPV	30.924	< 0.001	0.285	58.419	< 0.001	0.393	52.49	< 0.001	0.371
I will not vaccinate if the HPV vaccine is not mandatory	35.365	< 0.001	0.303	37.722	< .001	0.314	38.908	< 0.001	0.317
I will vaccinate against HPV regardless of the costs	48.102	< 0.001	0.355	97.068	< 0.001	0.505	81.644	< 0.001	0.462
All my closest friends are vaccinated against HPV	38.112	< 0.001	0.315	24.297	< 0.001	0.252	23.616	< 0.001	0.248
I would vaccinate against HPV if my doctor would recommend it	78.218	< 0.001	0.453				89.861	< 0.001	0.485
I would vaccinate if it was free of charge or with very little costs				78.218	< 0.001	0.453	100.529	< 0.001	0.51
I would vaccinate against HPV if I could get the vaccine in school	100.529	< 0.001	0.51	89.861	< 0.001	0.485			

Table S2. Gender comparison

Variable	Gender comparison		
	Chi2	p	Cramer's V
The HPV infection is an STD	3.409	0.182	0.131
The HPV infection can be asymptomatic	3.981	0.137	0.141
The use of condoms can prevent the HPV infection	4.105	0.128	0.144
The HPV infection can cause infertility	0.665	0.717	0.058
Genital warts or condylomas can be caused by HPV	3.103	0.212	0.126
HPV can cause Cervical Cancer	6.148	0.046	0.177
Mandatory vaccines can prevent children from getting infected by unvaccinated children	4.689	0.096	0.155
I understand what the HPV vaccine is used for	1.998	0.368	0.101
Genital warts or condylomas can make it harder to find a sexual partner	1.687	0.43	0.093
I'm more likely to trust older vaccines	1.398	0.497	0.084
Vaccines get better because of research	4.976	0.083	0.158
Children should only be vaccinated against serious diseases	5.477	0.065	0.166
Healthy children do not need to be vaccinated	0.493	0.781	0.05
An HPV vaccine could prevent future health issues	1.669	0.434	0.092
Administering the HPV vaccine for children is like experimenting on them	4.895	0.086	0.158
Most of the people I know believe that the HPV vaccine before adolescence is a good ideea	2.739	0.254	0.118
A teenager should be vaccinated against HPV without parental consent	0.509	0.775	0.051
Vaccine are very painful so I would prefer not to vaccinate against HPV	5.7	0.058	0.174
I will not vaccinate if the HPV vaccine is not mandatory	3.717	0.205	0.128
I will vaccinate against HPV regardlessof the costs	0.47	0.79	0.05
All my closest friends are vaccinated against HPV	4.142	0.126	0.147
I would vaccinate against HPV if my doctor would recommed it	0.534	0.776	0.053
I would vaccinate against HPV if it was free of charge or with very little costs	2.828	0.243	0.121
I would vaccinate against HPV if I could get the vaccine in school	0.878	0.645	0.067
